# Maternal Recognition of Ultrasound-Observed Fetal Movements Among Pregnancies in the Third Trimester: Concordance, Directional Discrepancies, and Associated Factors

**DOI:** 10.3390/medicina62102038

**Published:** 2026-10-21

**Authors:** Pattama Tongdee, Tasanun Nitipreecha, Porntip Nimkuntod, Atcharawan Rattanasak, Talit Jumphoo, Wongsathon Pathonsuwan, Kasidit Kokkhunthod, Sittinon Thanonklang, Monthippa Uthansakul, Peerapong Uthansakul

**Affiliations:** 1 School of Obstetrics and Gynecology, Institute of Medicine, Suranaree University of Technology, Nakhon Ratchasima 30000, Thailand; pattama_t@sut.ac.th (P.T.); tasanun@sut.ac.th (T.N.); 2 Suranaree University of Technology Hospital, Nakhon Ratchasima 30000, Thailand; 3 School of Internal Medicine, Institute of Medicine, Suranaree University of Technology, Nakhon Ratchasima 30000, Thailand; 4 School of Telecommunication Engineering, Suranaree University of Technology, Nakhon Ratchasima 30000, Thailand; d6500658@g.sut.ac.th (A.R.); m6700751@g.sut.ac.th (S.T.); mtp@sut.ac.th (M.U.); 5 Institute of Research and Development, Suranaree University of Technology, Nakhon Ratchasima 30000, Thailand; jumphoo@sut.ac.th (T.J.); pathonsuwan.wst@sut.ac.th (W.P.); kasidit234@gmail.com (K.K.)

**Keywords:** fetal movement, maternal perception, maternal recognition, temporal concordance, ultrasonography, gestational age, fetal monitoring

## Abstract

*Background and Objectives*: Maternal perception of fetal movement is widely used to monitor fetal activity; however, maternally perceived movements may not correspond directly to movements observed by ultrasonography. This study aimed to quantify same-window maternal recognition of ultrasound-observed fetal movements, characterize temporal concordance and directional discrepancies, and examine associated maternal and pregnancy-related factors. *Materials and Methods*: Pregnant women at ≥28 weeks of gestation underwent simultaneous 20 min fetal-movement assessment using real-time ultrasonography and maternal button-press reporting. The ultrasound observer pressed a designated button whenever fetal movement was visually observed, while participants independently pressed a separate button whenever they perceived fetal movement. Both responses were electronically timestamped using the same system clock and segmented into consecutive 5 s windows. Windows containing an ultrasound-observer button press were classified as ultrasound-positive, and those accompanied by a maternal response within the corresponding window were classified as temporally matched. Same-window maternal recognition was defined as the proportion of ultrasound-positive windows accompanied by a maternal response within the same 5 s analytical window. Associated factors were examined using fractional logistic regression with robust standard errors. *Results*: A total of 112 valid assessment sessions were analyzed. The median number of ultrasound-positive windows was 26.5 (IQR, 15.5–41.5), compared with 10.5 (IQR, 6.0–18.5) maternal-response windows. Median same-window maternal recognition was 20.3% (IQR, 8.3–38.5%; bootstrap percentile 95% CI, 15.7–23.1%), and median overall temporal concordance was 16.1% (IQR, 7.4–25.3%). Maternal-response window counts were lower than ultrasound-positive window counts in 101 of 112 assessment sessions (90.2%). After adjustment for maternal age, BMI, and fetal presentation, later gestational age was associated with lower same-window maternal recognition (β = −0.063, 95% CI −0.122 to −0.004; *p* = 0.037), corresponding to an average marginal effect of −1.12 percentage points per week (95% CI −2.15 to −0.09; *p* = 0.033). *Conclusions*: Maternal and ultrasound-observed fetal-movement counts showed limited same-window temporal correspondence during the 20 min observation period. A modest inverse association with gestational age was observed in the primary model, although the association was less precise in the denominator-aware and vertex-only sensitivity analyses. These findings reflect temporal matching under the predefined 5 s analytical framework and should not be interpreted as indicating that mothers perceive only a small proportion of fetal movements or that maternal perception becomes less reliable as pregnancy advances.

## 1. Introduction

Fetal movement is an important manifestation of fetal activity and neurological development and remains an integral component of antenatal assessment [1,2,3,4]. Maternal awareness provides a simple, non-invasive means of monitoring fetal activity, while reduced or altered fetal movement may prompt further clinical evaluation because such changes have been associated with adverse pregnancy outcomes, including late stillbirth [1,2,3,4].

Maternal perception, however, is subjective and does not necessarily represent all fetal motor activity occurring in utero. Several maternal and pregnancy-related factors have been proposed to influence perception, including gestational age, maternal body size, placental location, parity, maternal attention, psychological factors, and characteristics of individual fetal movements [5,6,7,8,9,10]. Evidence regarding these determinants remains inconsistent, and differences in study populations and movement definitions have contributed to heterogeneity in reported findings.

Real-time ultrasonography permits direct visualization of fetal motor activity and has therefore been used to investigate the correspondence between observed fetal movements and maternal perception. Earlier simultaneous studies consistently demonstrated incomplete maternal recognition [11,12,13,14,15,16]. Schmidt et al. reported differences in maternal perception according to movement type [11,12]. Hijazi et al. subsequently reported maternal perception of 35.8% of ultrasound-observed movements [14], whereas Brown et al. found a median recognition of 44.8%, with marked interindividual variability [16]. Recognition was more likely for movements involving multiple fetal body parts, movements of longer duration, and movements producing contact with the uterine wall [14,16].

More recently, Patel et al. performed a prospective ultrasound study using concurrent maternal perception and a 5 s temporal concordance criterion [17]. Maternal perception corresponded to 10.0% of ultrasound-observed movements at 32–34 weeks and 11.4% at 36–38 weeks [17]. These estimates, which are lower than those reported in several earlier studies, underscore the importance of considering the operational definition of temporal matching when comparing maternal recognition across studies. Short temporal matching windows may capture a different aspect of maternal perception than broader event-based or count-based approaches.

These observations highlight an important distinction between overall fetal-movement counts and temporal concordance. Maternal and ultrasound-observed movement counts may be correlated across assessment sessions even when individual maternal responses do not closely correspond in time to ultrasound-observed activity. Characterizing temporally matched windows together with ultrasound-only and maternal-only windows may therefore provide information beyond simple comparisons of total movement counts. Moreover, the direction and magnitude of discrepancies between maternal- and ultrasound-observed activity across different levels of fetal movement have received comparatively less attention.

Therefore, the primary objective of this study was to quantify same-window maternal recognition of ultrasound-observed fetal movements during prespecified simultaneous monitoring in pregnancies at ≥ 28 weeks of gestation. Secondary objectives were to characterize temporal concordance and directional discrepancies between maternal and ultrasound-observer recordings and to examine maternal and pregnancy-related factors associated with same-window maternal recognition.

## 2. Materials and Methods

### 2.1. Study Design and Participants

This observational study was conducted at the antenatal care (ANC) clinic of Suranaree University of Technology Hospital, Nakhon Ratchasima, Thailand, between September 2025 and June 2026. Pregnant women with a singleton pregnancy at ≥28 weeks of gestation were recruited according to predefined eligibility criteria. Women were excluded if they had underlying chronic medical conditions or if the fetus had known thoracic or abdominal organ abnormalities, including cardiac, pulmonary, gastrointestinal, or renal abnormalities. Eligible pregnant women attending the antenatal care clinic were approached consecutively and invited to participate in the study. Enrollment depended on participants’ availability and willingness to provide informed consent. Only women who agreed to participate were included in the study records. A comprehensive screening and recruitment log was not maintained; therefore, the total number of women approached, the number who declined participation, and the participation rate could not be determined retrospectively. All participants provided written informed consent before participation.

The original sample-size calculation was undertaken to support a planned evaluation of a newly developed fetal-movement detection device using ultrasonography as the reference assessment method. A one-sample proportion test was used, with an investigator-defined reference proportion of 70%. The anticipated proportion was set at 81% to represent an improvement of more than 10 percentage points above this reference value. These proportions were investigator-defined planning assumptions rather than estimates derived from previous empirical studies. With a two-sided significance level of 0.05 and 80% power, the calculated sample size was 100 participants. The recruitment target was increased to 110 participants to allow for anticipated data loss or incomplete assessments. All participants were aged 18 years or older at enrollment, and no minors were enrolled. The maternal age data were verified against the original source records, and a data-entry error affecting the recorded age of one participant was identified and corrected before the final analyses.

The original protocol did not specify an explicit operational formula for the proposed accuracy measure. Consequently, the original sample-size calculation cannot be directly linked to the session-level same-window maternal recognition outcome reported in the present manuscript. The calculation was based on participant recruitment rather than assessment sessions and was not designed to establish statistical power for the present recognition or associated-factor analyses. These analyses should therefore be interpreted as exploratory.

Fetal-movement assessments were conducted during prespecified 20 min simultaneous observation sessions in which fetal movements visualized by real-time ultrasonography and maternally perceived fetal movements were recorded simultaneously and independently. Maternal age, gestational age at assessment, body weight, height, body mass index (BMI), abdominal circumference, and fetal presentation were recorded at each assessment. Fetal presentation was categorized as vertex, breech, or transverse; for regression analyses, breech and transverse presentations were combined into a non-vertex category.

A total of 110 pregnant women were enrolled. Although repeated assessment was initially planned, a second assessment was feasible in only three participants, resulting in 113 assessment sessions. One assessment session was excluded following retrospective data-quality review. The recording showed an unusually high frequency of ultrasound-observer button signals (178 episodes, including 134 within the first 5 min), inconsistent with the fetal movements observed by the examiner. Technical review raised concerns regarding recording reliability, although the precise cause could not be confirmed. Because genuine button presses could not be reliably distinguished from potentially erroneous signals, the session was excluded based on a post hoc data-quality assessment rather than a prespecified numerical criterion. Consequently, 112 assessment sessions were retained for analysis.

### 2.2. Simultaneous Fetal Movement Assessment

Fetal movements were assessed simultaneously using real-time ultrasonography and maternal button-press reporting during a 20 min observation period. The 20 min observation period was prespecified in the study protocol and was selected to align with the conventional initial assessment period used for nonstress testing in antenatal fetal surveillance [18]. Ultrasound assessment was performed transabdominally using a portable SonoScape E2 ultrasound system (SonoScape Medical Corp., Shenzhen, China) equipped with a 3C-A convex-array transducer. Real-time ultrasonography was performed by one of two obstetricians, each with more than 7 years of experience in obstetric ultrasonography. Before data collection, the two obstetricians agreed on a predefined operational criterion for identifying fetal movement. A button press was recorded when definite fetal movement was visually observed, whereas isolated twitches or minimal movements were not recorded as fetal-movement events. Each button press generated an electronic timestamp that was subsequently used to identify ultrasound-observed fetal-movement events. For a continuous episode of fetal movement without clearly distinguishable individual movements, the ultrasound observer pressed the recording button once at the onset of the episode. When separate movements occurred in close succession, the observer pressed the button again for each distinguishable movement. The same recording procedure was consistently applied by both obstetricians throughout the study.

During the 20 min assessment, participants remained in the supine position and were positioned so that they could not view the ultrasound screen. Participants were instructed to press a separate response button whenever they perceived fetal movement and to press the button again when they perceived a subsequent distinguishable movement, including movements occurring in close succession. For a single prolonged episode of perceived fetal movement, participants were informed that they could press the response button repeatedly if the movement continued. However, no specific instructions were provided regarding the frequency or timing of repeated button presses during such episodes. Maternal responses were electronically timestamped by the same recording system. The ultrasound observer was blinded to maternal button-press responses during the assessment. Because both ultrasound-observer and maternal button presses were recorded using the same system clock, no additional synchronization of the two time series was required. The time of day of the assessment, recent maternal food intake, and recent maternal physical activity were not standardized or systematically recorded as part of the study protocol.

### 2.3. Signal Segmentation and Temporal Matching

The synchronized timestamp data were segmented into consecutive 5 s analytical windows using a preprocessing and labeling algorithm developed for the study. A window containing at least one button press by the ultrasound observer, indicating fetal movement visually observed during real-time ultrasonography, was classified as an ultrasound-positive window. A window without an ultrasound-observer button press was classified as ultrasound-negative.

Multiple fetal movements observed within the same 5 s window were not treated as separate analytical events; instead, the window was classified once as ultrasound-positive. If fetal movement extended across more than one 5 s window and an ultrasound-observer button press was recorded within each window, each corresponding window was classified as ultrasound-positive. Thus, the analytical unit was the 5 s window rather than an individual fetal movement.

Maternal button-press timestamps were mapped to the same 5 s analytical windows. An ultrasound-positive window containing a maternal button press was classified as a temporally matched window. An ultrasound-positive window without a maternal button press was classified as an ultrasound-only window, whereas a window containing a maternal button press without a corresponding ultrasound-observer button press was classified as a maternal-only window.

Temporal matching was defined strictly according to the predefined 5 s window boundaries. Consequently, an ultrasound-observed movement and a maternal response occurring in adjacent windows were not classified as matched, even if they occurred close to the shared window boundary. The resulting measure therefore represents same-window maternal–ultrasound temporal correspondence rather than event-level confirmation of maternal perception.

### 2.4. Definition of Study Outcomes

The primary outcome was same-window maternal recognition of ultrasound-observed fetal movement, defined as the proportion of ultrasound-positive windows accompanied by a maternal response within the same 5 s analytical window:

Same-window maternal recognition (%) = (temporally matched windows/ultrasound-positive windows) × 100

Secondary descriptive measures included the numbers of ultrasound-positive windows, maternal-response windows, temporally matched windows, ultrasound-only windows, and maternal-only windows.

Overall temporal concordance was calculated as:

Overall temporal concordance (%) = temporally matched windows/(temporally matched windows + ultrasound-only windows + maternal-only windows) × 100

This measure represents the proportion of all movement-positive windows identified by either source in which both the ultrasound observer and the mother recorded a fetal-movement response within the same 5 s analytical window.

### 2.5. Directional Discrepancy Between Maternal and Ultrasound Counts

Directional discrepancy was evaluated by comparing the total number of maternal-response windows with the total number of ultrasound-positive windows within each assessment session. The session-level difference was calculated as:

Difference = maternal-response window count − ultrasound-positive window count

Negative values indicated fewer maternal-response than ultrasound-positive windows, positive values indicated more maternal-response than ultrasound-positive windows, and a value of zero indicated equal window counts from the two sources within that assessment session.

### 2.6. Statistical Analysis

Continuous variables were summarized as mean ± standard deviation (SD) or median and interquartile range (IQR), as appropriate according to their distributions. For the primary descriptive outcome, the median same-window maternal recognition was additionally reported with a 95% confidence interval estimated using percentile bootstrap resampling with 20,000 replications. Categorical variables were summarized as frequencies and percentages. The distributions of maternal-response and ultrasound-positive window counts and their paired differences were examined before analysis. Because the paired differences were non-normally distributed, maternal-response and ultrasound-positive window counts were compared using the Wilcoxon signed-rank test. The association between maternal-response and ultrasound-positive window counts was assessed using Spearman’s rank correlation coefficient.

Agreement between maternal-response and ultrasound-positive window counts was explored using Bland–Altman analysis. The session-level difference was defined as the maternal-response window count minus the ultrasound-positive window count, and the mean of the two window counts was used as the horizontal-axis measure. The mean difference and conventional 95% limits of agreement (mean difference ± 1.96 SD of the differences) were calculated descriptively. Because the distribution of differences deviated from normality, the conventional limits of agreement were interpreted descriptively. Evidence of proportional bias was assessed by examining the relationship between the paired differences and their means using Spearman’s rank correlation and linear regression with robust standard errors.

Same-window maternal recognition was analyzed as a fractional outcome ranging from 0 to 1. Associations between maternal and pregnancy-related characteristics and same-window maternal recognition were examined using fractional logistic regression with a logit link and robust standard errors. The multivariable model included maternal age, gestational age, BMI, and fetal presentation (vertex versus non-vertex). Results are presented as regression coefficients (β) with 95% confidence intervals (CIs) and *p*-values. Average marginal effects (AMEs) were additionally calculated to facilitate interpretation of associations on the probability scale.

Sensitivity analyses were performed to evaluate the robustness of the association between gestational age and same-window maternal recognition. First, the primary model was repeated after restricting the analysis to assessment sessions conducted at ≤38 weeks of gestation. Second, a quadratic term for gestational age was added to evaluate potential nonlinearity. Third, abdominal circumference was substituted for BMI as an alternative measure of maternal body size. Fourth, to account for variation in the number of ultrasound-positive windows contributing to each recognition proportion, a denominator-aware sensitivity analysis was performed using a grouped-binomial logistic model, with the number of temporally matched windows specified as successes and the number of ultrasound-positive windows as trials. The model included the same covariates as the primary model and used robust standard errors. Fifth, to examine whether the association between gestational age and same-window maternal recognition was influenced by overall ultrasound-observed fetal-movement activity, the number of ultrasound-positive windows was added to the primary fractional logistic model. Sixth, the primary model was repeated after restricting the analysis to assessment sessions with vertex presentation. Seventh, an exploratory inclusion sensitivity analysis was performed by reinstating the assessment session excluded during the post hoc data-quality review and repeating the primary multivariable fractional logistic regression model using all 113 assessment sessions. Because participant-level identifiers linking the small number of repeated assessments were not available in the analytical dataset, within-participant correlation could not be explicitly modeled. All statistical tests were two-sided, and *p* < 0.05 was considered statistically significant. Analyses were performed using Stata version 19.5 (StataCorp LLC, College Station, TX, USA).

### 2.7. Ethical Considerations

This study was conducted in accordance with the Declaration of Helsinki and was approved by the Human Research Ethics Committee, Suranaree University of Technology (COA No. 215/2567). Written informed consent was obtained from all participants before enrollment and before any study-specific procedures were performed.

## 3. Results

### 3.1. Participant and Pregnancy Characteristics

A total of 112 valid assessment sessions with complete paired maternal and ultrasound fetal-movement data were included in the final analysis. Maternal age at assessment ranged from 18 to 39 years, with a mean of 29.88 ± 5.03 years. Mean gestational age at assessment was 33.41 ± 3.26 weeks (range, 28–40 weeks). Mean maternal BMI was 29.21 ± 5.56 kg/m^2^, and mean abdominal circumference was 104.42 ± 9.51 cm.

Vertex presentation was recorded in 96 of 112 assessment sessions (85.7%), whereas breech and transverse presentations were recorded in 11 (9.8%) and 5 (4.5%) sessions, respectively. For subsequent analyses, breech and transverse presentations were combined into a non-vertex category (16/112, 14.3%). Maternal and pregnancy characteristics at the analyzed assessment sessions are summarized in Table 1.

### 3.2. Maternal- and Ultrasound-Observed Fetal-Movement Activity

Ultrasound-positive fetal-movement windows were more frequent than maternal-response windows. The median number of ultrasound-positive windows per assessment session was 26.5 (IQR, 15.5–41.5), compared with 10.5 (IQR, 6.0–18.5) maternal-response windows. The median number of temporally matched windows was 5.0 (IQR, 2.0–10.0), whereas the median numbers of ultrasound-only and maternal-only windows were 20.0 (IQR, 11.5–32.0) and 6.0 (IQR, 3.0–10.0), respectively (Table 2).

Maternal-response window counts were lower than ultrasound-positive window counts in 101 of 112 assessment sessions (90.2%), higher in 10 (8.9%), and identical in 1 (0.9%). The paired distributions differed significantly (Wilcoxon signed-rank test, z = −8.406, *p* < 0.001).

The median same-window maternal recognition was 20.3% (IQR, 8.3–38.5%; bootstrap percentile 95% CI, 15.7–23.1%). The median overall temporal concordance was 16.1% (IQR, 7.4–25.3%).

Despite the difference in their distributions, maternal-response and ultrasound-positive window counts were moderately correlated (Spearman’s ρ = 0.510, *p* < 0.001).

### 3.3. Maternal Recognition and Temporal Concordance

Same-window maternal recognition varied considerably across assessment sessions, ranging from 0% to 80.0%, with a mean of 23.4 ± 19.1%. The median was 20.3% (IQR, 8.3–38.5%; bootstrap percentile 95% CI, 15.7–23.1%). This measure reflects maternal–ultrasound temporal correspondence within the predefined 5 s analytical windows and should not be interpreted as the proportion of all individual fetal movements perceived by the mother.

No temporally matched windows were observed in 12 of 112 assessment sessions (10.7%). In three assessment sessions (2.7%), no maternal-response windows were recorded despite the presence of ultrasound-positive fetal-movement windows.

When temporally matched, ultrasound-only, and maternal-only windows were considered jointly, the median overall temporal concordance was 16.1% (IQR, 7.4–25.3%), indicating limited temporal overlap between maternal responses and ultrasound-observer recordings within the predefined 5 s analytical windows.

### 3.4. Directional Discrepancy and Exploratory Agreement Analysis

The session-level difference between maternal-response and ultrasound-positive window counts was calculated as the maternal-response count minus the ultrasound-positive count. The mean difference was −15.95 windows (SD, 16.11), indicating that maternal-response window counts were, on average, lower than ultrasound-positive window counts. The conventional 95% limits of agreement were −47.53 to 15.64 windows. Given the non-normal distribution of the paired differences, these limits were interpreted descriptively.

The magnitude of the discrepancy varied across the range of fetal-movement activity. The maternal–ultrasound difference was inversely correlated with the mean of the two window counts (Spearman’s ρ = −0.428, *p* < 0.001). Consistent with this finding, robust linear regression showed that the maternal–ultrasound difference decreased by 0.447 windows for each one-window increase in the mean of the maternal-response and ultrasound-positive counts (β = −0.447, 95% CI −0.729 to −0.165; *p* = 0.002).

**Figure 1:**
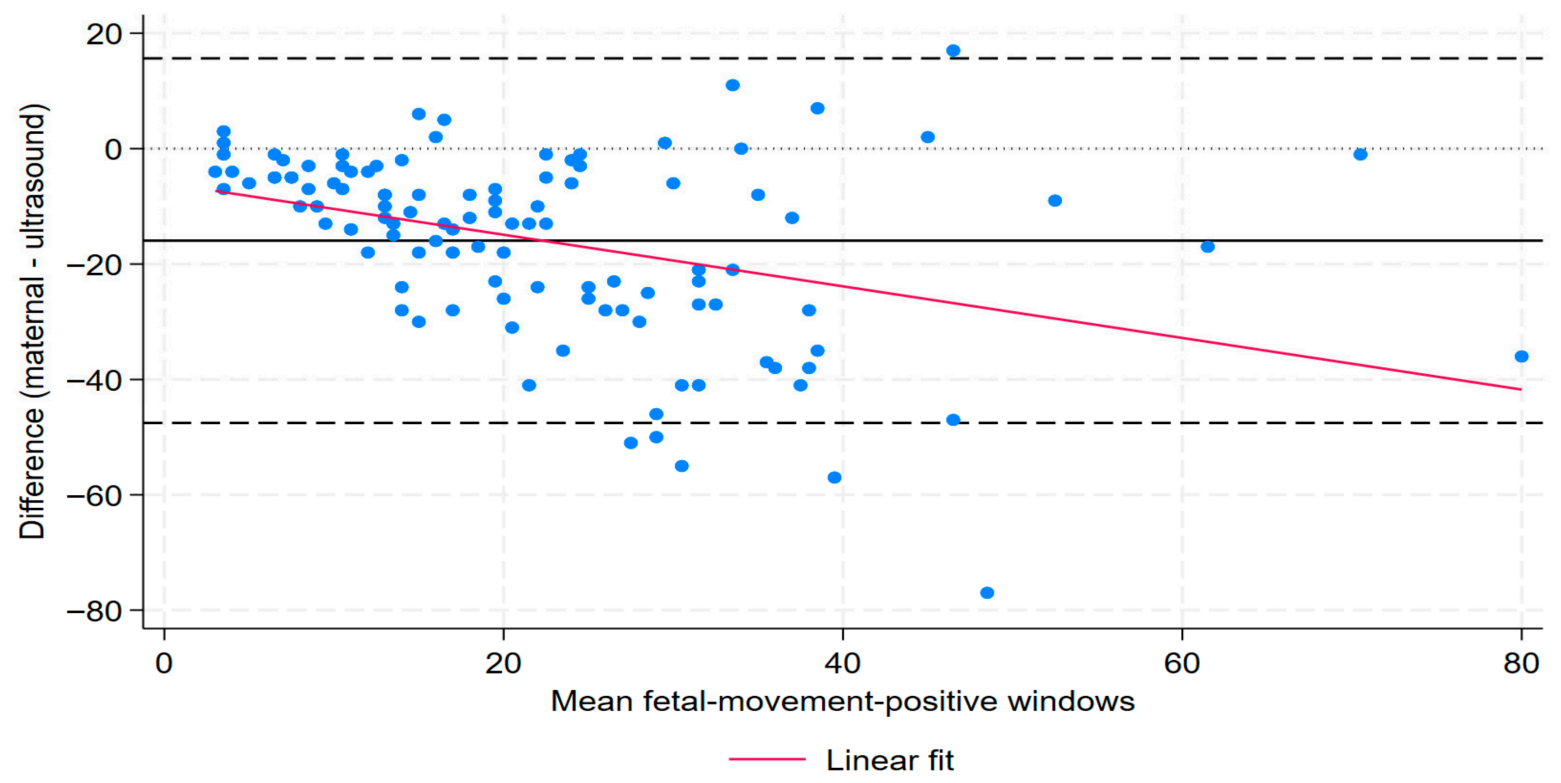
Bland–Altman plot of maternal-response and ultrasound-positive fetal-movement window counts. The difference was calculated as the maternal-response window count minus the ultrasound-positive window count and plotted against the mean of the maternal-response and ultrasound-positive window counts. The solid horizontal line represents the mean difference (−15.95 windows), the dashed lines represent the conventional 95% limits of agreement (−47.53 to 15.64 windows), and the dotted horizontal line represents zero difference. The fitted linear regression line illustrates the relationship between the paired difference and the mean window count.

These findings were consistent with proportional bias, with increasingly negative maternal–ultrasound differences as the mean fetal-movement window count increased (Figure 1).

### 3.5. Unadjusted Associations with Maternal Recognition

In unadjusted analyses, maternal recognition was inversely correlated with gestational age (Spearman’s ρ = −0.249, *p* = 0.008). In contrast, no clear correlations were observed between maternal recognition and maternal age (ρ = 0.094, *p* = 0.325), BMI (ρ = 0.024, *p* = 0.802), or abdominal circumference (ρ = 0.011, *p* = 0.912).

Gestational age was not clearly associated with the number of ultrasound-positive windows (Spearman’s ρ = −0.153, *p* = 0.108). Similarly, in a linear regression model with robust standard errors, the estimated change was −0.72 ultrasound-positive windows per additional week of gestation (95% CI, −1.87 to 0.43; *p* = 0.219).

Maternal recognition did not differ significantly between assessment sessions with vertex and non-vertex fetal presentations (Wilcoxon rank-sum *p* = 0.897).

Overall temporal concordance showed a similar pattern. It was inversely correlated with gestational age (ρ = −0.263, *p* = 0.005), whereas no clear correlations were observed with maternal age (ρ = 0.115, *p* = 0.227), BMI (ρ = 0.029, *p* = 0.759), or abdominal circumference (ρ = 0.007, *p* = 0.939). Overall temporal concordance also did not differ significantly between assessment sessions with vertex and non-vertex fetal presentations (Wilcoxon rank-sum *p* = 0.917).

### 3.6. Multivariable Factors Associated with Same-Window Maternal Recognition

Fractional logistic regression was used to examine maternal and pregnancy-related factors associated with same-window maternal recognition. The prespecified multivariable model included maternal age, gestational age, BMI, and fetal presentation (vertex versus non-vertex).

After adjustment, gestational age was inversely associated with same-window maternal recognition (β = −0.0630, 95% CI −0.122 to −0.004; *p* = 0.037). No clear associations were observed for maternal age, BMI, or non-vertex fetal presentation (Table 3).

Average marginal effects were calculated to facilitate interpretation on the probability scale. Each additional week of gestation was associated with an estimated 1.12-percentage-point lower same-window maternal recognition (AME = −0.0112, 95% CI −0.0215 to −0.0009; *p* = 0.033).

The corresponding AMEs were 0.0029 (95% CI −0.0044 to 0.0102; *p* = 0.436) for maternal age, 0.0011 (95% CI −0.0053 to 0.0075; *p* = 0.735) for BMI, and −0.0238 (95% CI −0.1189 to 0.0712; *p* = 0.623) for non-vertex versus vertex presentation.

### 3.7. Sensitivity Analyses

First, when the analysis was restricted to assessment sessions conducted at ≤38 weeks of gestation (*n* = 108), the inverse association between gestational age and same-window maternal recognition persisted (β = −0.066, 95% CI −0.127 to −0.006; *p* = 0.032). The corresponding average marginal effect was −0.0119 (95% CI −0.0225 to −0.0013; *p* = 0.028), equivalent to an estimated 1.19-percentage-point lower same-window maternal recognition per additional week of gestation.

Second, potential nonlinearity was examined by including a quadratic term for gestational age in the model. The quadratic term was not statistically significant (β = −0.005, 95% CI −0.027 to 0.016; *p* = 0.614), with no clear evidence of departure from linearity within the observed gestational-age range.

Third, abdominal circumference was substituted for BMI in an alternative multivariable model to assess the robustness of the findings to an alternative measure of maternal body size. Gestational age remained inversely associated with same-window maternal recognition (β = −0.065, 95% CI −0.125 to −0.005; *p* = 0.034), whereas no clear association was observed between abdominal circumference and same-window maternal recognition (β = 0.005, 95% CI −0.018 to 0.029; *p* = 0.654).

Fourth, a denominator-aware sensitivity analysis was performed using a grouped-binomial logistic model in which the number of temporally matched windows was specified as the number of successes and the number of ultrasound-positive windows as the number of trials. The association between gestational age and same-window maternal recognition was similar in direction and magnitude to that observed in the primary fractional logistic model, although the statistical evidence was weaker (β = −0.067, 95% CI −0.137 to 0.003; *p* = 0.062).

Fifth, to examine whether the association with gestational age could be explained by variation in overall ultrasound-observed fetal-movement activity, the number of ultrasound-positive windows was added to the primary fractional logistic model. The association between gestational age and same-window maternal recognition remained similar (β = −0.067, 95% CI −0.128 to −0.006; *p* = 0.031), corresponding to an AME of −0.0119 (95% CI −0.0225 to −0.0013; *p* = 0.028). The number of ultrasound-positive windows was not clearly associated with same-window maternal recognition (β = −0.004, 95% CI −0.016 to 0.008; *p* = 0.493).

Sixth, in an analysis restricted to assessment sessions with vertex presentation (*n* = 96), the association between gestational age and same-window maternal recognition remained inverse in direction but was less precise (β = −0.049, 95% CI −0.112 to 0.013; *p* = 0.121). The corresponding AME was −0.0088 (95% CI −0.0197 to 0.0021; *p* = 0.115).

Seventh, an exploratory sensitivity analysis including the excluded assessment session (*n* = 113) yielded an adjusted association between gestational age and same-window maternal recognition similar to that observed in the primary analysis (*n* = 112): β = −0.0626 (95% CI −0.1222 to −0.0031; *p* = 0.039) versus β = −0.0630 (95% CI −0.1224 to −0.0037; *p* = 0.037), respectively.

Overall, the direction of the association between gestational age and same-window maternal recognition was broadly consistent across alternative model specifications and sensitivity analyses. However, statistical evidence was weaker in the denominator-aware model and in the vertex-only analysis, in which the confidence intervals included the null.

## 4. Discussion

In this observational study of pregnancies in the third trimester, maternal responses accompanied only a proportion of fetal-movement-positive windows identified during real-time ultrasonography. Although maternal-response and ultrasound-positive window counts were moderately correlated, temporal concordance between the two sources was relatively low. Maternal-response counts were lower than ultrasound-positive counts in most assessment sessions, demonstrating a predominantly directional discrepancy. In addition, later gestational age was associated with modestly lower same-window maternal recognition in the primary multivariable model, whereas maternal age, BMI, and fetal presentation showed no clear associations. Together, these findings highlight the distinction between overall correlation in fetal-movement counts and same-window maternal–ultrasound temporal correspondence.

### 4.1. Interpretation of Maternal Recognition and Temporal Concordance

The present study demonstrated limited same-window temporal correspondence between maternal responses and ultrasound-observed fetal movement, with a median same-window maternal recognition of 20.3%. This estimate should be interpreted within the predefined 5 s window-based analytical framework rather than as the proportion of all individual fetal movements perceived by the mother. Previous simultaneous ultrasound studies have also reported incomplete temporal correspondence between maternal perception and ultrasound-observed fetal movement, although estimates have varied considerably according to study design and matching criteria [11,12,13,14,15,16,17]. Schmidt et al. reported that mothers perceived approximately 38% of fetal movements identified by real-time ultrasonography [11], whereas Hijazi et al. reported maternal perception of 35.8% of 763 ultrasound-observed fetal movements [14]. Brown et al. demonstrated marked interindividual variability, with women perceiving 2.4–81.0% of ultrasound-observed movements and a median recognition of 44.8% [16]. More recently, Patel et al. reported lower recognition estimates using concurrent maternal perception and a 5 s temporal concordance criterion, with maternal perception corresponding to 10.0% of ultrasound-observed movements at 32–34 weeks and 11.4% at 36–38 weeks [17].

Direct numerical comparisons across these studies should, however, be made cautiously because their analytical definitions differ. In the present study, synchronized recordings were divided into consecutive 5 s analytical windows, and maternal perception was considered temporally matched only when a maternal response occurred within the same window as an ultrasound-observer response. Multiple movements occurring within a single 5 s window were treated as one ultrasound-positive analytical unit. Consequently, the 20.3% recognition estimate in the present study represents temporal correspondence within a predefined window-based framework rather than the proportion of all individual fetal movements perceived by the mother. Furthermore, because temporal matching was based on fixed, non-overlapping 5 s windows, an ultrasound-observed movement and a maternal response occurring close to a shared window boundary could be classified as unmatched despite close temporal proximity. Accordingly, the recognition estimate should be regarded as a measure of same-window maternal–ultrasound temporal correspondence under the prespecified analytical framework.

An additional finding was the coexistence of moderate correlation between maternal-response and ultrasound-positive window counts (Spearman’s ρ = 0.510) and relatively low overall temporal concordance (median, 16.1%). These measures capture different aspects of correspondence. Correlation indicates whether assessment sessions with greater ultrasound-observed fetal activity also tend to contain more maternal responses, whereas temporal concordance reflects whether maternal and ultrasound-observer responses occur within the same predefined analytical window. Thus, moderate correlation in overall activity can coexist with limited same-window temporal correspondence.

### 4.2. Directional Discrepancy Between Maternal- and Ultrasound-Observed Fetal Movement

Maternal-response window counts were lower than ultrasound-positive window counts in 101 of 112 assessment sessions (90.2%), indicating that the discrepancy was predominantly directional rather than random. The mean maternal–ultrasound difference was −15.95 windows, although the wide conventional limits of agreement indicated substantial between-session variability. Furthermore, increasingly negative differences at higher mean fetal-movement window counts were consistent with proportional bias, suggesting that the magnitude of the discrepancy increased during sessions with greater fetal-movement activity.

This pattern is biologically plausible and is consistent with previous evidence that maternal perception depends partly on the characteristics of individual fetal movements [11,12,13,14,15,16]. Earlier ultrasound studies demonstrated that movements involving multiple fetal body parts were more likely to be perceived than isolated movements [11,12,13]. Brown et al. similarly found that synchronous trunk-and-limb movements were more frequently perceived than movements involving either component alone and that longer-duration movements were more likely to be recognized [16]. These observations provide one possible explanation for why ultrasonography may identify subtle or localized fetal activity without a corresponding maternal response. In addition, closely spaced or continuous movements may be experienced by the mother as a single episode while generating ultrasound-observer responses across multiple analytical windows [11,12,13,14,15,16].

The Bland–Altman findings should, however, be interpreted as exploratory. Because the paired differences were non-normally distributed and evidence of proportional bias was present, the conventional limits of agreement should not be interpreted as establishing interchangeability between maternal-response and ultrasound-positive window counts. Rather, this analysis provides complementary information regarding the direction and magnitude of discrepancies across different levels of fetal-movement activity.

### 4.3. Gestational Age and Maternal Recognition

Later gestational age was associated with modestly lower same-window maternal recognition in the primary multivariable model, corresponding to an estimated 1.12-percentage-point lower recognition per additional week of gestation. The direction of this association was generally consistent across sensitivity analyses, including restriction to assessments at ≤38 weeks, substitution of abdominal circumference for BMI, and adjustment for the number of ultrasound-positive windows. However, the association was less precise in the denominator-aware grouped-binomial model and in the vertex-only analysis, with confidence intervals that included the null. In addition, the quadratic analysis showed no clear evidence of departure from linearity within the observed gestational-age range. Taken together, these findings suggest a modest inverse association, but its magnitude and statistical certainty were sensitive to analytical specification.

Previous studies have not demonstrated a consistent relationship between gestational age and maternal perception of fetal movement [5,11,17,19]. Hertogs et al. found no clear association between gestational age and maternal awareness of fetal movement [19], whereas Schmidt et al. reported greater maternal perception of fetal body movements with advancing gestation [11]. A subsequent review concluded that evidence regarding the influence of gestational age on maternal perception remained conflicting [5]. More recently, Patel et al., using a 5 s temporal concordance criterion, reported similar maternal perception proportions at 32–34 and 36–38 weeks [17].

The inverse association observed in the primary model should therefore be interpreted cautiously. Although the direction of the association was generally consistent across sensitivity analyses, the denominator-aware and vertex-only analyses yielded less precise estimates with confidence intervals that included the null. Moreover, the present study was primarily cross-sectional, with only a small number of participants contributing repeated assessments; therefore, these findings do not demonstrate a longitudinal decline in an individual woman’s ability to perceive fetal movement as gestation advances. The absence of a clear association between gestational age and the number of ultrasound-positive windows also suggests that the primary finding was not simply explained by lower ultrasound-observed fetal-movement activity at later gestational ages, although residual confounding and model-dependent uncertainty remain possible. Changes in fetal-movement characteristics across late gestation could potentially contribute to the observed pattern, but movement type, duration, and intensity were not measured; therefore, mechanistic explanations cannot be established from the present data.

### 4.4. Maternal Characteristics and Fetal Presentation

Maternal age, BMI, and fetal presentation showed no clear associations with maternal recognition in the adjusted analysis. Similar findings were obtained when abdominal circumference was substituted for BMI as an alternative measure of maternal body size. Previous studies have reported inconsistent associations between maternal characteristics and fetal-movement perception [5,6,16,20,21]. Hertogs et al. found no clear associations of maternal awareness with obesity, parity, or placental location [19], and Brown et al. reported no significant associations between maternal perception and BMI, parity, placental location, or amniotic fluid index during simultaneous ultrasound monitoring [16]. Similarly, a systematic review found limited and very-low-quality evidence that increased maternal body size itself impairs perception of fetal movement [20].

Other studies, however, have reported associations under different clinical or analytical conditions. For example, anterior placental location and nulliparity have been associated with presentation for reduced fetal movement [6], while a large prospective study examining the onset of maternal perception found that increasing BMI, advancing maternal age, nulliparity, and anterior placental position were associated with later first perception of fetal movement [21]. More recently, Patel et al. reported associations of maternal age and BMI with maternal perception during concurrent ultrasound assessment [17]. These differences emphasize that determinants of initial perception, presentation with reduced fetal movement, and temporally matched recognition during simultaneous ultrasound monitoring represent related but distinct outcomes.

The absence of statistically significant associations in the present study should not be interpreted as evidence of no effect. In particular, only 16 assessment sessions involved non-vertex presentation, resulting in limited precision for comparisons by fetal presentation. Furthermore, fetal presentation is a relatively broad anatomical descriptor and does not capture the spatial relationships among the moving fetal part, placenta, uterine wall, and maternal abdominal wall. Future studies incorporating detailed fetal position and placental location may provide a more complete assessment of anatomical influences on maternal perception. Parity was not available in the retained analytical dataset and therefore could not be evaluated as a potential maternal factor in the present analysis. Given previous evidence suggesting that parity may influence maternal perception of fetal movement, this should be considered when interpreting the adjusted findings.

### 4.5. Clinical Interpretation

The findings of this study should not be interpreted as diminishing the clinical importance of maternal awareness of fetal movement. Maternal perception remains an accessible and clinically relevant component of antenatal surveillance, and reduced or altered fetal movement may warrant further clinical assessment because it is associated with adverse pregnancy outcomes, including late stillbirth [1,2,3,4]. Current guidance recommends that women report a decrease or cessation of fetal movements to their maternity care provider [1].

The relatively low same-window temporal concordance observed in this study does not imply that maternal perception is unreliable or clinically uninformative. Maternal awareness reflects the mother’s experience of her fetus over time and may incorporate changes in the frequency, strength, and pattern of movements that cannot be represented by a single 20 min observation period. Conversely, real-time ultrasonography provides direct visualization of fetal activity during a defined period but does not reproduce the mother’s longitudinal experience of her fetus. The two approaches therefore capture different aspects of fetal-movement assessment [1,22].

From a clinical perspective, the present findings support distinguishing maternal awareness of changes in the fetus’s usual movement pattern from one-to-one temporal detection of movements visualized by ultrasonography. A maternal report of reduced or altered fetal movement should continue to prompt appropriate clinical assessment according to established obstetric guidance [1]. However, evidence that formal fetal-movement counting or interventions designed to increase awareness reduce stillbirth remains uncertain [23,24,25,26]. The present study was not designed to evaluate diagnostic accuracy or pregnancy outcomes and should not be used to define a numerical threshold for clinically important fetal movement or to replace existing clinical assessment pathways.

### 4.6. Strengths and Limitations

This study has several strengths. Fetal movements were assessed prospectively using simultaneous real-time ultrasonography and maternal button-press reporting, with both responses electronically timestamped using the same system clock. This design minimized uncertainty arising from post hoc temporal synchronization and enabled same-window maternal recognition to be evaluated using a predefined 5 s analytical window. The ultrasound observer was blinded to maternal button-press responses during the assessment, reducing the potential for observer responses to be influenced by maternal reporting. In addition, the study distinguished among temporally matched, ultrasound-only, and maternal-only windows, allowing temporal concordance and directional discrepancies to be examined separately from overall movement-count correlation.

Several limitations should also be considered. First, the study was conducted at a single antenatal clinic, which may limit the generalizability of the findings to populations with different maternal, obstetric, or healthcare characteristics. Although eligible women were approached consecutively, enrollment depended on their availability and willingness to participate. Because a comprehensive screening and recruitment log was not maintained, the number of women who declined participation and the participation rate could not be determined. Selection bias therefore cannot be excluded. Second, each assessment lasted 20 min and therefore represents only a limited sampling period of fetal activity. Maternal perception in routine clinical practice reflects fetal-movement patterns experienced over much longer periods. Third, ultrasound-observed fetal movement was identified in real time by one of two experienced obstetricians using a predefined operational criterion, whereby definite fetal movements were recorded and isolated twitches or minimal movements were not recorded as fetal-movement events. Although both observers had more than 7 years of experience in obstetric ultrasonography and used the same predefined criterion, fetal-movement identification remained observer dependent. Formal interobserver reproducibility was not assessed; therefore, observer-related variability cannot be excluded.

Fourth, temporal matching relied on fixed, non-overlapping 5 s analytical windows. Consequently, an ultrasound-observed movement and a maternal response occurring close to a shared window boundary could be classified as unmatched despite close temporal proximity. Recognition estimates may therefore differ if alternative temporal windows, shifted-window approaches, or event-based definitions are used. Because raw event-level timestamps were not retained in the final analytical dataset, alternative event-centered or shifted-window sensitivity analyses could not be performed. For continuous fetal-movement episodes, the ultrasound observer recorded a single button press at movement onset rather than continuously marking the duration of the episode. Consequently, subsequent 5 s windows during an ongoing movement episode might not have been classified as ultrasound-positive in the absence of additional button presses. The resulting estimates therefore reflect temporal correspondence between recorded responses and may not fully capture the duration of continuous fetal activity. Accordingly, direct numerical comparisons with studies using different temporal definitions should be made cautiously [14,16,17,22]. One assessment session was excluded following a post hoc data-quality review because of concerns regarding the reliability of ultrasound-observer button recordings. Although the recorded signal pattern was inconsistent with the examiner’s observations, the precise technical cause could not be confirmed. The exclusion criterion was not prespecified. An exploratory sensitivity analysis including the excluded session did not materially alter the principal association. However, the validity of the questionable recording could not be established, and the post hoc exclusion remains a methodological limitation.

Fifth, movement characteristics such as type, duration, intensity, and the number of fetal body parts involved were not separately classified, although previous studies suggest that these characteristics influence maternal perception [14,16]. Placental location was also not available for analysis [5,6,21]. Parity was also not available in the retained analytical dataset and could not be included as a potential covariate. Residual confounding by parity or other unmeasured maternal and obstetric characteristics therefore cannot be excluded. In addition, the time of day of the assessment, recent maternal food intake, and recent maternal physical activity were neither standardized nor systematically recorded. Potential variation in these factors may have influenced fetal activity or maternal perception during the observation period and could not be accounted for in the analyses.

Sixth, three participants underwent an additional assessment approximately one month after their initial assessment. Because participant-level identifiers linking these repeated assessments were not retained in the analytical dataset, potential within-participant correlation could not be explicitly modeled. However, the number of additional repeated assessments was small relative to the overall analytical dataset. An additional limitation concerns the original sample-size calculation, which was developed for a planned device-accuracy evaluation rather than the session-level same-window maternal recognition outcome analyzed in the present study. Because the original protocol did not specify an operational formula for the proposed accuracy measure, the calculation cannot be directly linked to the current outcome. Therefore, the present recognition and associated-factor analyses should be interpreted as exploratory. Finally, the sample size provided limited precision for some subgroup comparisons, particularly non-vertex presentation, which was present in only 16 assessment sessions. In the vertex-only sensitivity analysis, the gestational-age association remained inverse in direction but was less precise and its confidence interval included the null. Accordingly, the absence of statistically significant associations in subgroup or sensitivity analyses should not be interpreted as evidence of no association.

### 4.7. Future Research

Future studies should validate these findings in larger and more diverse populations and should retain participant-level identifiers to permit appropriate modeling of repeated assessments. Longer or repeated monitoring periods may help determine whether temporal recognition varies within individuals across gestation. Detailed characterization of fetal movement type, duration, intensity, fetal position, and placental location would also help clarify factors influencing maternal perception [5,14,16,22].

Methodological studies comparing alternative temporal matching windows and event-based definitions may be particularly valuable, as recognition estimates appear sensitive to the analytical framework used [14,16,17,22]. Emerging wearable and sensor-based technologies may also enable longer and more objective monitoring of fetal activity outside short ultrasound observation periods [27,28]. Future studies should determine whether such technologies can be validated against clinically meaningful reference measures and whether temporal recognition patterns are associated with pregnancy outcomes. Such work may help clarify whether quantitative measures of maternal–ultrasound correspondence provide information beyond maternal awareness of changes in usual fetal-movement patterns.

## 5. Conclusions

Maternal responses and ultrasound-observed fetal movements showed limited same-window temporal correspondence during simultaneous 20 min monitoring in the third trimester. Maternal-response and ultrasound-positive window counts were moderately correlated, but same-window temporal concordance within the predefined 5 s analytical framework was relatively low, and maternal-response counts were lower than ultrasound-positive counts in most assessment sessions. In the primary multivariable model, later gestational age showed a modest inverse association with same-window maternal recognition; however, this association was less precise in the denominator-aware and vertex-only sensitivity analyses. These findings reflect temporal matching under the predefined analytical framework and should not be interpreted as indicating that mothers perceive only a small proportion of all fetal movements or that maternal perception becomes less reliable as pregnancy advances. Further studies using alternative temporal-matching approaches and longitudinal designs are needed to determine the clinical significance of maternal–ultrasound temporal correspondence.

## Figures and Tables

**Table 1:** Maternal and pregnancy characteristics at the analyzed assessment sessions (*n* = 112).

Characteristic	Value
Maternal age, years	29.88 ± 5.03
Gestational age, weeks	33.41 ± 3.26
Weight, kg	73.03 ± 14.07
Height, cm	158.19 ± 5.39
BMI, kg/m^2^	29.21 ± 5.56
Abdominal circumference, cm	104.42 ± 9.51
Fetal presentation	
Vertex	96 (85.7)
Non-vertex	16 (14.3)
Breech	11 (9.8)
Transverse	5 (4.5)

Note: Data are based on 112 valid assessment sessions and are presented as mean ± SD or *n* (%), as appropriate. Non-vertex presentation comprised breech and transverse presentations and was used in subsequent regression analyses. BMI, body mass index; SD, standard deviation.

**Table 2:** Fetal-movement measures and maternal recognition across assessment sessions (*n* = 112).

Measure	Median (IQR)
Ultrasound-positive windows	26.5 (15.5–41.5)
Maternal-response windows	10.5 (6.0–18.5)
Temporally matched windows	5.0 (2.0–10.0)
Ultrasound-only windows	20.0 (11.5–32.0)
Maternal-only windows	6.0 (3.0–10.0)
Total discordant windows	28.0 (17.0–43.0)
Same-window maternal recognition, %	20.3 (8.3–38.5)
Ultrasound-only proportion, %	79.7 (61.5–91.7)
Overall temporal concordance, %	16.1 (7.4–25.3)

Note: Data are presented as median (IQR) across 112 valid assessment sessions. Total discordant windows comprised ultrasound-only plus maternal-only windows. Same-window maternal recognition was calculated as temporally matched windows divided by ultrasound-positive windows. The ultrasound-only proportion was calculated as ultrasound-only windows divided by ultrasound-positive windows. Overall temporal concordance was calculated as temporally matched windows divided by the sum of temporally matched, ultrasound-only, and maternal-only windows. IQR, interquartile range.

**Table 3:** Fractional logistic regression of factors associated with same-window maternal recognition (*n* = 112).

Variable	Coefficient (95% CI)	*p*-Value	AME (95% CI)	*p*-Value
Maternal age, per year	0.016 (−0.025 to 0.057)	0.436	0.0029 (−0.0044 to 0.0102)	0.436
Gestational age, per week	−0.063 (−0.122 to −0.004)	0.037	−0.0112 (−0.0215 to −0.0009)	0.033
BMI, per kg/m^2^	0.006 (−0.030 to 0.042)	0.735	0.0011 (−0.0053 to 0.0075)	0.735
Non-vertex vs. vertex	−0.134 (−0.667 to 0.400)	0.623	−0.0238 (−0.1189 to 0.0712)	0.623

Note: Coefficients (β) were estimated using fractional logistic regression with a logit link and robust standard errors. AMEs represent the average change in the predicted same-window maternal recognition proportion associated with a one-unit increase in each continuous variable or the change from vertex to non-vertex presentation for the categorical variable. BMI, body mass index; CI, confidence interval; AME, average marginal effect.

## Data Availability

The data supporting the findings of this study are available from the corresponding author upon reasonable request, subject to applicable ethical and institutional requirements. The data are not publicly available due to privacy and ethical considerations related to research involving human participants.
